# RNA quality in frozen breast cancer samples and the influence on gene expression analysis – a comparison of three evaluation methods using microcapillary electrophoresis traces

**DOI:** 10.1186/1471-2199-8-38

**Published:** 2007-05-22

**Authors:** Carina Strand, Johan Enell, Ingrid Hedenfalk, Mårten Fernö

**Affiliations:** 1 Lund University, Department of Oncology, Clinical Sciences, Lund, SE 221 85 Lund, Sweden

## Abstract

**Background:**

Assessing RNA quality is essential for gene expression analysis, as the inclusion of degraded samples may influence the interpretation of expression levels in relation to biological and/or clinical parameters. RNA quality can be analyzed by agarose gel electrophoresis, UV spectrophotometer, or microcapillary electrophoresis traces, and can furthermore be evaluated using different methods. No generally accepted recommendations exist for which technique or evaluation method is the best choice. The aim of the present study was to use microcapillary electrophoresis traces from the Bioanalyzer to compare three methods for evaluating RNA quality in 24 fresh frozen invasive breast cancer tissues: 1) Manual method = subjective evaluation of the electropherogram, 2) Ratio Method = the ratio between the 28S and 18S peaks, and 3) RNA integrity number (RIN) method = objective evaluation of the electropherogram. The results were also related to gene expression profiling analyses using 27K oligonucleotide microarrays, unsupervised hierarchical clustering analysis and ontological mapping.

**Results:**

Comparing the methods pair-wise, Manual *vs*. Ratio showed concordance (good *vs*. degraded RNA) in 20/24, Manual *vs*. RIN in 23/24, and Ratio *vs*. RIN in 21/24 samples. All three methods were concordant in 20/24 samples. The comparison between RNA quality and gene expression analysis showed that pieces from the same tumor and with good RNA quality clustered together in most cases, whereas those with poor quality often clustered apart. The number of samples clustering in an unexpected manner was lower for the Manual (n = 1) and RIN methods (n = 2) as compared to the Ratio method (n = 5).

Assigning the data into two groups, RIN ≥ 6 or RIN < 6, all but one of the top ten differentially expressed genes showed decreased expression in the latter group; *i.e*. when the RNA became degraded. Ontological mapping using GoMiner (p ≤ 0.05; ≥ 3 genes changed) revealed deoxyribonuclease activity, collagen, regulation of cell adhesion, cytosolic ribosome, and NADH dehydrogenase activity, to be the five categories most affected by RNA quality.

**Conclusion:**

The results indicate that the Manual and RIN methods are superior to the Ratio method for evaluating RNA quality in fresh frozen breast cancer tissues. The objective measurement when using the RIN method is an advantage. Furthermore, the inclusion of samples with degraded RNA may profoundly affect gene expression levels.

## Background

The development of high-throughput technologies such as microarrays, allowing for the parallel analysis of the expression of thousands of genes from a tumor in one single experiment, has provided new tumor biological knowledge. In breast cancer, for example, microarrays have been suggested to be useful for predicting clinical outcome and for tailoring treatment strategies for individual patients [1-3]. This approach may also increase the ability to identify new targets for more specific therapies. Studies using this technique have furthermore revealed differences in gene expression profiles between different subgroups of breast cancer, *e.g*. between hereditary and sporadic breast cancer, and between estrogen receptor (ER) positive and ER negative tumors [1,4,5].

Microarrays were first described by Schena and co-workers in 1995 [6]. The different parts of this technique involve RNA extraction, control of RNA quality, hybridization, and data analysis. Extraction of RNA is a long process, often in the presence of contaminants and ribonucleases that may degrade RNA. RNA is sensitive and can hence easily be degraded at room temperature. The most common technique for controlling the quality of RNA is the characterization with agarose gel electrophoresis and/or using a UV spectrophotometer. However, these techniques are not sensitive enough and are easily influenced by contaminants in the sample. Therefore new techniques have been developed, *e.g*. the Agilent 2100 Bioanalyzer [7]. The Bioanalyzer is based on a lab-on-a-chip micro-fluids technology, and the software generates an electropherogram and a gel-like image. With this new technique data can be evaluated in different ways, either manually by inspecting the electropherogram, or by calculating the 28S/18S ratio. Recently a new feature in the Bioanalyzer software has been implemented, the RNA integrity number (RIN) [8,9]. Furthermore, Auer and co-workers have developed a mathematical model for quantitative characterization of RNA degradation, the Degradometer [10]. No generally accepted recommendations exist, however, regarding which technique or evaluation method is the best choice for downstream applications requiring high quality RNA. Moreover, to our knowledge, no study has previously systematically evaluated to what extent the RNA quality influences the interpretation of gene expression profiling for routinely collected frozen breast cancer samples.

In the present study we have focused on 1) different ways of evaluating the quality of RNA, 2) how the quality of RNA influences microarray-based gene expression analyses, and 3) which type of gene categories that are affected by decreased RNA quality.

The results indicate that the Manual and RIN methods are superior to the Ratio method for evaluating RNA quality in fresh frozen breast cancer tissues. The objectively obtained measurement of the RIN method is, in addition, clearly an advantage. Furthermore, the inclusion of samples with degraded RNA can profoundly influence gene expression profiles, and hence clustering of samples as well as absolute expression levels of individual genes.

## Results

### RNA quality

**Figure 1:**
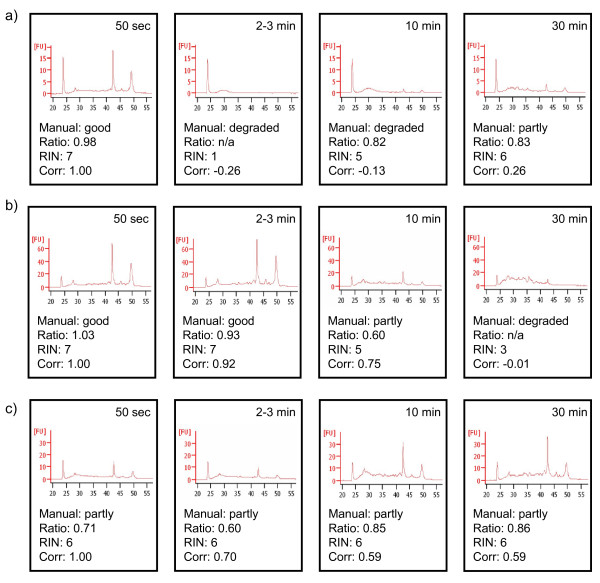
**Bioanalyzer electropherograms**. Bioanalyzer electropherograms of three samples, a) Sample 3, b) Sample 5, and c) Sample 6 after different lengths of time: 50 seconds, 2–3 minutes, 10 minutes, and 30 minutes, respectively. Three methods were used for evaluating the RNA quality, see Methods. Pearson correlation coefficients were obtained, when the gene expression levels of the sample for the different time periods at room temperature were related to the gene expression levels of the sample left at room temperature for 50 seconds.

We analyzed the RNA quality using three different methods; Manual, Ratio and RIN, respectively (see Methods). Visual inspection of the Bioanalyzer electropherograms showed that of the six samples included, the majority were degraded at room temperature, but after different lengths of time [see Additional file 1]. Three examples are shown in Fig. 1. Based on manual evaluation, the Manual method, Sample 3 was degraded at 2 minutes, Sample 5 at 30 minutes, whereas Sample 6 was not affected at all within the 30 minute time-frame (Fig. 1a–c).

**Figure 2:**
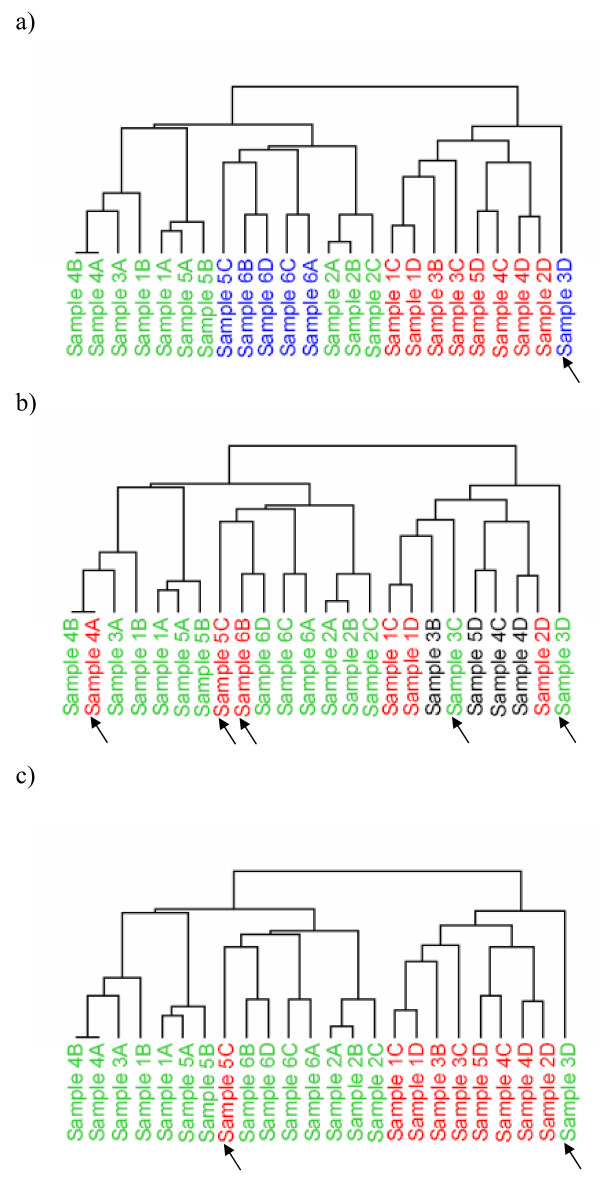
**Unsupervised hierarchical clustering**. Unsupervised hierarchical clustering was used to assess which samples clustered together based on their gene expression profiles. A. Clustering according to the Manual evaluation method; green = good, blue = partly degraded, red = degraded. B. Clustering according to the Ratio method; green = ratio ≥ 0.65 (*i.e*. good), red = ratio < 0.65 (*i.e*. degraded), and black = N/A (*i.e*. not available). C. Clustering according to the RIN method; green = RIN ≥ 6 and red = RIN < 6. Arrows indicate samples clustering in an unexpected manner, according to the respective methods.

**Figure 3:**
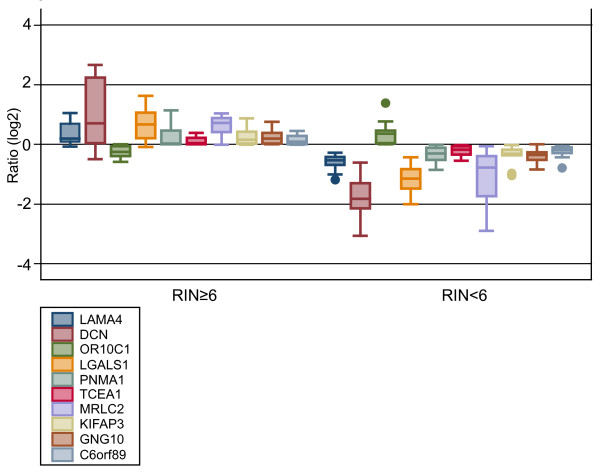
**Top ten differentially expressed genes**. The top ten most differentially expressed genes between RIN ≥ 6 and RIN < 6. *LAMA4 *= laminin 4, *DCN *= decorin, *OR10C1 *= olfactory receptor, *LGALS1 *= lectin galactoside-binding, *PNMA1 *= paraneoplastic antigen, neuron and testis specific protein, *TCEA1 *= transcription elongation factor A, *MRLC2 *= myosin regulatory light chain, *KIFAP3 *= kinesin-associated protein 3, *GNG10 *= guanine nucleotide binding protein, and *C6orf89 *= chromosome 6 open reading frame 89. Filled circles represent outliers.

Similar results were obtained using the Ratio method. According to the Ratio method however, Sample 3 was considered good at 10 minutes (Fig. 1a), whereas Samples 5 and 6 were considered poor at 10 minutes (Fig. 1b) and 2–3 minutes (Fig. 1c), respectively. The RIN method, on the other hand, yielded results almost identical to the Manual method. One exception was, however, noted; Sample 5 (Fig. 1b, 10 min) was considered good with the Manual method, but not with the RIN method.

The electropherograms from one of the samples (Sample 3), showed an unexpected appearance over time (Fig. 1a). It was degraded at 2 and 10 minutes, but at 30 minutes, the RNA was considered partly degraded with the Manual method and good with the Ratio and RIN methods.

In summary, pair-wise comparisons of the methods revealed that Manual *vs*. Ratio showed concordance in 20/24, Manual *vs*. RIN in 23/24, and Ratio *vs*. RIN in 21/24 samples. All three methods showed concordant results in 20 of the 24 samples.

### Gene expression

Our hypothesis was that if the RNA quality of the sample was good for all four time periods, the corresponding gene expression profiles should be similar and the samples should consequently cluster together. Conversely, upon RNA degradation, changes in gene expression profiles would cause the sample replicates to cluster apart. Using unsupervised hierarchical clustering to assess which samples clustered together, we noted that the samples clustered into two separate groups, one including most of the good samples (including those partly degraded) and one including most of the degraded samples, irrespective of evaluation method (Fig. 2).

When using the Manual method, all samples but one (Fig. 2a, arrow) clustered as we hypothesized. The corresponding number of samples, clustering in an unexpected way with the other two methods (*i.e*. samples considered to be of good RNA quality clustering with degraded samples or *vice versa) *was five (Ratio method; Fig. 2b) and two (RIN method; Fig. 2c), respectively.

Concentrating on RIN values we assigned the data into two groups: RIN ≥ 6 or RIN < 6 and compared the gene expression profiles of these two groups to see whether there was a significant difference for any given reporter × between the two groups. We performed a gene score analysis in Bio Array Software Environment (BASE) [11] to find statistical significance in terms of false discovery rates (FDR), and a permutation test was performed to obtain an estimate of the rate of differentially expressed reporters. Out of 14,288 reporters, 7,672 distinguished the two groups with an FDR of 5%. With an FDR of 0.01%, 238 reporters were able to distinguish between the two groups. The top ten most differentially expressed genes are shown in Fig. 3. All but one showed decreased gene expression levels in the RIN < 6 compared to the RIN ≥ 6 group. Similar results were obtained when a t-test and a Mann-Whitney test were used to calculate probabilities (data not shown).

Gene Ontology (GO) mapping using GoMiner [12], (p ≤ 0.05 and ≥ 3 changed genes in each category) revealed deoxyribonuclease activity, GO: 0004536 (12%); collagen, GO: 0005581 (9.1%); regulation of cell adhesion, GO: 0030155 (8.3%); cytosolic ribosome (sensu Eukaryota), GO: 0005830 (7.3%); and NADH dehydrogenase activity GO: 0003954 (6.7%) to be the five most affected categories, [see Additional file 2].

## Discussion

Good RNA quality is essential for obtaining reliable result from microarray experiments. The inclusion of samples with degraded RNA may influence the statistical analysis and hence the interpretation of gene expression levels in relation to biological and/or clinical data. Results should reflect true biological differences and not differences due to poor RNA integrity.

In the present study, three different evaluation methods were compared, one manual and two objective (the Ratio and RIN methods). In 20/24 (83%) samples, all three methods came to the same result (good or degraded RNA). The Manual and RIN methods were concordant in 23/24 (96%) samples, whereas the Ratio method showed discordant results with the other two methods in four and three samples, respectively. In some of the discordant samples, the discrepancy could be explained by values near the cut-off. The results indicate that the Manual and RIN methods are more similar to one another than the Ratio method is to either. This finding is in line with the evaluation principles. While both the Manual and RIN methods take the whole electropherogram into consideration, manually or objectively, the Ratio method relies only on the ratio between the 28S and 18S peaks. Furthermore, the ratio calculation is based on area measurements and is heavily dependent on the definitions of the start and end of the peaks. In addition, small peaks make this measurement even more uncertain, which is often the case with partly degraded samples. Therefore, the ribosomal ratio may not be sufficient to evaluate RNA degradation efficiently in all instances. Copois and co-workers, using colorectal cancer, liver metastases, and normal colon, compared the ratio method with the computer-based RIN and Degradometer methods, as well as with an in-house "RNA Quality Scale" method, and came to the conclusion that the 28S/18S ratio resulted in misleading categorization [13]. To address this issue, Sotiriou and co-workers used an arbitrary cut-off of 15% of the total RNA area, and 28S/18S > 1.1 in their investigation of the correlation between histological grade and gene expression profiles in breast cancer [14].

Imbeaud and co-workers obtained similar results in their study including both cell lines and different normal tissues, demonstrating ambiguity with the Ratio method [15]. When ribosomal ratios were calculated from identical samples, a large degree of variability was observed. Manual evaluation of the RNA quality through visual inspection, on the other hand, provided consistent data. In general, there was a good agreement between the manual classification, the degradation factor and the RIN method, but not with the ratio values [15].

In concordance with the above-mentioned studies, the results of our investigation demonstrate that the gene expression profiles change considerably upon RNA degradation. We hypothesized that if the RNA quality in different samples from the same breast tumor was good, the corresponding gene expression profiles should be similar, and the samples should consequently cluster together. In contrast, when RNA is degraded, changes in gene expression profiles would cause the samples to cluster apart. Our findings indicate that the results of the RNA quality evaluation using the Manual and RIN methods were more concordant with the results of the clustering analyses than when using the Ratio method. While only one (Manual) and two (RIN) sample replicates clustered apart, five samples clustered in an unexpected way when the Ratio method was used, *i.e*. samples considered to be of good RNA quality clustered with degraded samples or *vice versa*. These results indicate that the Manual and RIN methods are more concordant and superior to the Ratio method for evaluating RNA quality in fresh frozen breast cancer samples. An advantage with the RIN method in comparison with the Manual method is that it yields an objective measurement, whereas the subjective interpretation of the Manual method, especially for the partly degraded group, may show both intra- and inter-individual variation. In order to validate the cut-off of 6 for the RIN method, we also tested 5 or 7 as cut-offs. The number of samples clustering in an unexpected way was thereby increased to three and seven, respectively. The use of 6 as a cut-off was also strengthened when the RIN values were compared to the Pearson correlation coefficients of the association between the gene expression of the samples for the different time points (2–3 minutes to 50 minutes) and the gene expression after 50 seconds.

One sample showed an unexpected appearance over time, as the RNA quality appeared superior after extended exposure to room temperature compared to shorter time periods when it was deemed degraded (Fig. 1a). This surprising observation may be explained by tumor heterogeneity.

Of the top ten most differentially expressed genes, all but one showed decreased gene expression levels in the RIN < 6 compared to the RIN ≥ 6 group (Fig. 3), suggesting degradation of RNA transcripts to occur as RNA quality deteriorates. Furthermore, gene ontology mapping using GoMiner revealed deoxyribonuclease activity, collagen, regulation of cell adhesion, cytosolic ribosome, and NADH dehydrogenase activity to be the five categories most affected by RNA quality. One may speculate that genes belonging to these categories could potentially be used as markers for RNA quality in gene profiling studies using fresh frozen breast cancer tissue. It would be interesting to evaluate if this strategy could be used as a potential qualification approach for already collected gene expression data sets, and to investigate whether clustering analyses are influenced due to the inclusion of degraded transcripts belonging to these ontological categories.

Our results demonstrate that RNA was degraded at room temperature, but the RNA in the six samples showed variable sensitivity. This variation may be explained by different sensitivity to room temperature due to *e.g*. differences in tissue composition. Some samples may be rich in fatty tissue, whereas others may be rich in epithelial cancer cells. Furthermore, the amount of connective tissue may also influence the amount and quality of extracted RNA. Another explanation for the differences between samples may be that the time period from surgical excision until the sample is placed at -80°C varies and that they are collected from several pathological departments, with different routines. The tissue composition and suboptimal sample collection procedures may also explain the relatively low ratio values obtained in breast cancer, in comparison with other tissue materials. In a recent publication from our group [16], we had approximately the same percentage of samples with poor RNA quality (9%) in comparison with other studies using similar criteria for evaluation of the RNA quality (10–20%) [17,18].

From the electropherograms it was, furthermore, demonstrated that RNA degradation is a gradual process. Not all RNA follows the same pattern during degradation; however, the larger ribosome is typically degraded first, resulting in a decrease and broadening of this peak. Consequently, as degradation proceeds, there is a decrease in the 28S to 18S ribosomal ratio and an increase in the baseline signal between the two ribosomal peaks.

## Conclusion

The results indicate that the Manual and RIN methods are superior to the Ratio method for evaluating RNA quality in fresh frozen breast cancer tissues. The RIN method gives an objective measure of RNA quality, while the Manual method may be subject to inter-, as well as intra-observer variation. In addition, the inclusion of samples with degraded RNA may affect the outcome of the study, as the levels of gene expression are highly dependent upon RNA integrity. Based on our experience, we recommend RIN values ≥ 6 to be used for fresh frozen breast cancer tissue.

## Methods

### Study design

Frozen samples from six patients were retrieved from the tissue bank (-80°C) owned by the South Swedish Breast Cancer Group. In order to obtain RNA of different quality, four equally sized pieces (by weight) from each invasive breast cancer sample were placed at room temperature for four different lengths of time: 50 seconds, 2–3 minutes, 10 minutes, and 30 minutes, after which the samples were placed in liquid nitrogen.

The ethical committee at Lund University approved this project.

### RNA isolation and quality control

The samples were pulverized with a Micro-dismembrator II (B. Braun Biotech Int., Germany), and RNA was extracted using Trizol reagent (Invitrogen, Carlsbad, CA), and purified with Qiagen RNeasy Midi columns (Qiagen, Chatsworth, CA). The RNA concentration was determined using a Nanodrop Spectrophotometer (NanoDrop Technologies, Wilmington, DE). The RNA quality was assessed using an Agilent 2100 Bioanalyzer (Agilent Technologies, Santa Clara, CA) together with the reagents in the RNA 6000 Nano LabChip kit. All samples were within the kit capacity (5–500 ng/μl). The Agilent 2100 Bioanalyzer generates an electropherogram and a gel-like image and displays results such as sample RNA concentration and the so called ribosomal ratio, *i.e*. the ratio between the ribosomal subunits, 28S/18S.

The electropherogram can be evaluated in three ways. With visual inspection, (Manual method) the quality of RNA is considered good if the electropherogram shows two distinct peaks, one for 28S and one for 18S, and a flat baseline (*e.g*. Fig. 1a, 50 sec.). The electropherogram of a degraded sample contains many small peaks and a highly elevated baseline (*e.g*. Fig. 1b, 30 min.). In addition to the good and degraded are the partly degraded samples; two peaks are visible, but the baseline is elevated (*e.g*. Fig. 1c, 50 sec.). Most of these are considered good enough for further analysis, *i.e*. to proceed to the hybridization step. However, methods that rely on visual inspection are subjective and have a tendency to vary over time. A more objective way to evaluate the quality of RNA may be to use a certain threshold for the 28S/18S ratio as a cut-off (Ratio method). From previous studies, we have established a threshold for the Bioanalyzer ratio at ≥ 0.65 (data not shown). A more recent approach is to use the RNA Integrity Number (RIN) method, which is a standardization of RNA quality control [8,19]. It is a software algorithm that has been developed to extract information about RNA sample integrity from Bioanalyzer electrophoretic trace. The RIN method was developed to eliminate the effect of individual interpretation on RNA quality control. It takes the entire electropherogram into consideration and is based on a numbering system from 1 to 10, where 1 represents the most degraded RNA and 10 represents intact RNA. When the RIN tool was developed, input data included approximately 1,300 total RNA samples from various tissues, all with varying levels of RNA integrity [19]. After a threshold value has been established, this value can be used in the RNA quality control procedure, but if any experimental parameter is changed (*e.g*. type of organism, type of tissue, type of microarray platform, RNA extraction procedure, *etc.) *the validation procedure needs to be repeated. There are, thus, no established cut-off values and each laboratory needs to establish their own.

Previously, we have compared RIN values with results from the Manual method in a series of 163 breast tumors, used in other projects. In these projects the samples were extracted in, essentially, the same way as in the present study. All samples considered to be of good RNA quality with the Manual method had RIN values between 6 and 8 (median 7). The median values for the partly degraded and degraded were 6 (range: 3–7) and 4 (range: 2–6), respectively. Based on these results we considered values greater or equal to 6 to represent good RNA. This cut-off was therefore also used in the present study.

### cDNA microarrays

Five micrograms of tumor RNA was labeled with Cy3^® ^dCTP (Amersham Biosciences, Piscataway, NJ), and 5 μg of reference RNA (Stratagene, La Jolla, CA), consisting of a pool of ten different tumor cell lines, was labeled with Cy5^® ^dCTP (Amersham Biosciences, Piscataway, NJ), according to the manufacturer's instructions using the reagents in the ChipShot™ labeling system kit (Corning Inc., Corning, NY).

Arrays were produced by the Swegene DNA Microarray Resource Centre, Department of Oncology at Lund University, Sweden, using a set of 26,819 70 base-pair human oligonucleotide probes (Operon Ver. 2.1. and Ver 2.1.1 upgrade, Cat.No. 810516 and 810518), which were obtained from Operon Biotechnologies, Inc. (Huntsville, AL). The probes represent 16,641 gene symbols.

Prior to hybridization, slides were UV-cross linked at 800 mJ/cm^2 ^and pre-treated using the Pronto!™ Plus System 6 (Corning, Inc., Corning, NY), according to the manufacturer's instructions. Arrays were scanned at two wavelengths using an Agilent G2505A DNA microarray scanner (Agilent Technologies, Santa Clara, CA), with 10 μm resolution. Gene Pix Pro 4.0 software (Axon Instruments, Inc., Union City, CA), was used for image analysis. Gene names were linked to the spots and spots with poor quality were manually excluded. Raw-data are available at Gene Expression Omnibus [20].

### Data analysis

Background correction of Cy3 and Cy5 intensities was calculated, using the median feature and the median local background intensities provided in the data matrix. Within arrays, intensity ratios for individual features were calculated as background corrected intensity of tumor sample divided by background corrected intensity of reference sample. The data matrix was uploaded to BASE [11], where the data analysis took place.

Spots with intensities lower than zero, and spots that were flagged bad or not found were excluded. Reporters that were not present in 100% of the arrays were filtered out, and the data was normalized using Lowess [21], resulting in 14,288 reporters in the final analysis. Unsupervised hierarchical clustering, using Euclidean distance, was performed in BASE. Concentrating on RIN values, we assigned the data into two groups: RIN ≥ 6 or RIN < 6, and compared the gene expression profiles of these two groups to see whether there was a significant difference for any given reporter × between the two groups. We performed a gene score analysis in BASE to find statistical significance in terms of false discovery rates (FDR), and a permutation test was performed to obtain an estimate of the rate of differentially expressed reporters.

Ontological mapping using the publicly available software GoMiner [12] was performed to investigate the most significantly affected GO categories. A p-value ≤ 0.05 was used, and only categories with ≥ 3 changed genes were considered in the analysis. A percentage of the number of genes that were changed in each category was calculated.

### Validating the RIN threshold value

**Figure 4:**
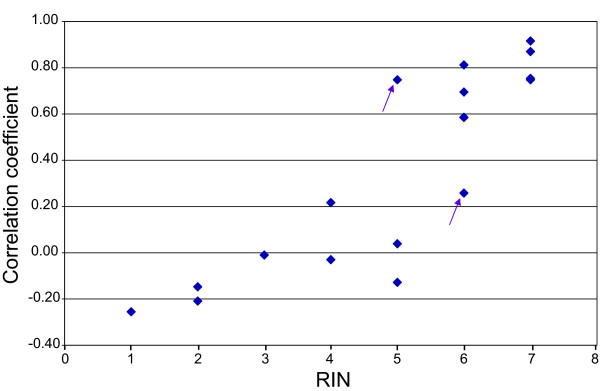
**Correlation between the RIN value and Pearson correlation**. In order to evaluate the RIN cut-off value of 6, we compared it to Pearson correlation coefficients. Pearson correlation coefficients were obtained, when the gene expression levels of the samples for the different time periods at room temperature, were related to the gene expression levels of the samples left 50 seconds at room temperature. If the correlation coefficient of the gene expression level is the true value, two samples obtain unexpected RIN values, *i.e*. a low correlation coefficient and a RIN value above the cut-off or *vice versa *(arrows, RIN 5 = Sample 5, 10 min and RIN 6 = Sample 3, 30 min).

In order to validate the RIN cut-off value, the RIN values were compared to the Pearson correlation coefficients (Fig. 4). In BASE, Pearson correlation coefficients were obtained, when the gene expressions of the sample for the different time points at room temperature were related to the gene expression of the sample left 50 seconds at room temperature. Poor correlations should correspond to lower RIN values, and good correlations should equal higher RIN values (Fig. 4). If the correlation coefficient of the gene expression is the true value for the RNA quality, only two samples did not obtain RIN values as expected, *i.e*. a low correlation coefficient and a RIN value above the cut-off or *vice versa *(Fig. 4). Both samples had a RIN value close to the cut-off. This strengthened the choice of 6 as the cut-off for the RIN method in the present study.

## Authors' contributions

MF conceived of the study. CS contributed to the development of methodology and executed the experiments. CS and JE analyzed and interpreted the data. CS, MF and IH took active part in writing the manuscript. All authors read and approved the final manuscript.

## Supplementary Material

Additional file 1 Bioanalyzer electropherograms. Bioanalyzer electropherograms for the six samples at different time points. Click here for file

Additional file 2 GO categories. Gene ontology analysis of the 7,672 differentially expressed genes using GoMiner, with a p-value ≤ 0.05 and with ≥ 3 changed genes in each category. Click here for file
