# Supplementary material for: RNA quality in frozen breast cancer samples and the influence on gene expression analysis – a comparison of three evaluation methods using microcapillary electrophoresis traces
Source: BMC Mol Biol. 2007 May 22;8:38. doi: 10.1186/1471-2199-8-38 (PMC1891312; doi:10.1186/1471-2199-8-38)
Supplement: Additional file 1 — Bioanalyzer electropherograms. Bioanalyzer electropherograms for the six samples at different time points. [file 1471-2199-8-38-S1.pdf]

	50 sec	2-3 min	10 min	30 min

a, Sample 1, 18269 — good | good | degr | degr

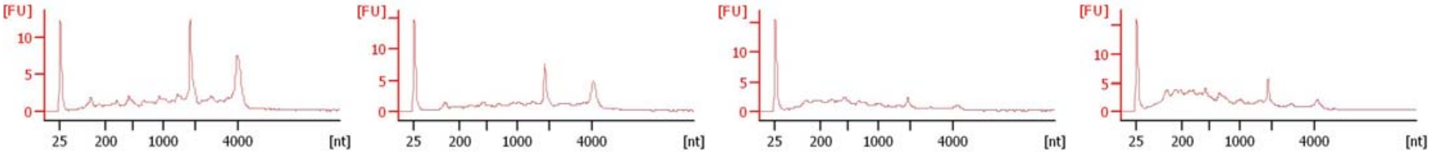

	50 sec	2-3 min	10 min	30 min
Ratio	1.04	1.01	0.54	0.44
RIN	7	7	4	5
Corr.	1.00	0.75	-0.03	0.04

b, Sample 2, 18245 — good | good | good | degr

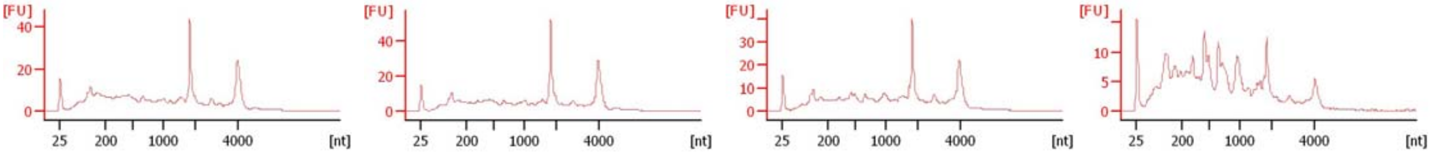

	50 sec	2-3 min	10 min	30 min
Ratio	1.04	1.06	1.56	0.60
RIN	6	7	6	4
Corr.	1.00	0.87	0.81	0.22

c, Sample 3, 18230 — good | degr | degr | partly

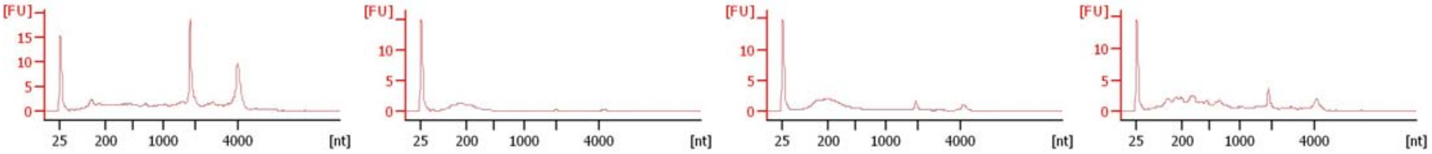

	50 sec	2-3 min	10 min	30 min
Ratio	0.98	n/a	0.82	0.83
RIN	7	1	5	6
Corr.	1.00	-0.26	-0.13	0.26

d, Sample 4, 18193 — good | good | degr | degr

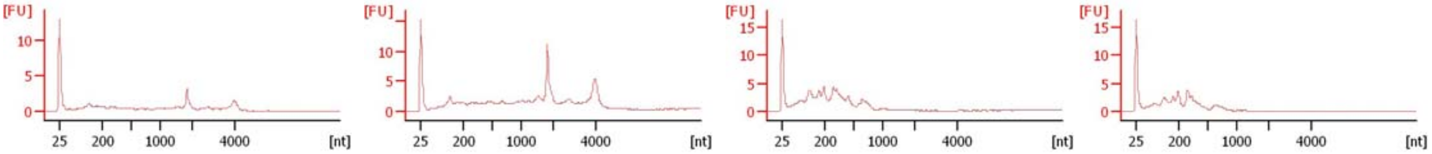

	50 sec	2-3 min	10 min	30 min
Ratio	0.49	0.72	n/a	n/a
RIN	7	7	2	2
Corr.	1.00	0.75	-0.15	-0.21

e, Sample 5, 18165 — good | good | partly | degr

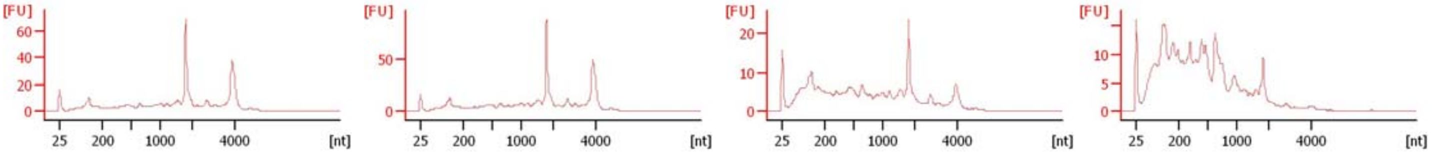

	50 sec	2-3 min	10 min	30 min
Ratio	1.03	0.93	0.60	n/a
RIN	7	7	5	3
Corr.	1.00	0.92	0.75	-0.01

f, Sample 6, 18146 — partly | partly | partly | partly

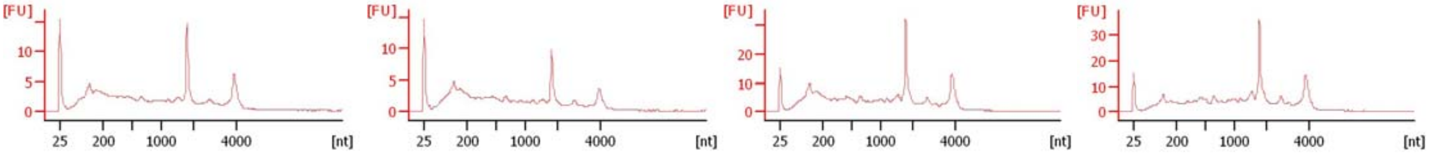

	50 sec	2-3 min	10 min	30 min
Ratio	0.71	0.60	0.85	0.86
RIN	6	6	6	6
Corr.	1.00	0.70	0.59	0.59
